# Activity‐aggression behavioural syndromes exist in males but not in females of the field cricket *Teleogryllus emma*

**DOI:** 10.1002/ece3.10642

**Published:** 2023-10-18

**Authors:** Chang S. Han, Byeongho Lee, Jong‐yeol Moon

**Affiliations:** ^1^ Department of Biology Kyung Hee University Seoul Korea

**Keywords:** animal personality, behavioural syndrome, field cricket, sex difference

## Abstract

Previous studies on sex differences in behaviour have largely focused on differences in average behaviours between sexes. However, males and females can diverge not only in average behaviours but also in the direction of behavioural correlations at the individual level (i.e. behavioural syndromes). Behavioural syndromes, with their potential to constrain the independent evolution of behaviours, may play a role in shaping sex‐specific responses to selection and contributing to the development of sex differences in behaviour. Despite the pivotal role of behavioural syndromes in the evolution of sexual dimorphism in behaviour, robust empirical evidence of sex differences in behavioural syndromes based on repeated measurements of behaviours is scarce. In this study, we conducted repeated measurements of activity and aggression in male and female field crickets *Teleogryllus emma*, providing evidence of sex differences in the existence of behavioural syndromes. Males exhibited a significantly positive behavioural syndrome between activity and aggression, whereas females, in contrast, did not show any aggressive behaviour, resulting in the absence of such a syndrome. The sex differences in the existence of the activity‐aggression behavioural syndromes in this species could be attributed to differences in selection. Selection favouring more active and aggressive males may have shaped a positive activity‐aggression behavioural syndrome in males, whereas the absence of selection favouring female aggression may have resulted in the absence of aggression and the related behavioural syndrome in females. However, given the plasticity of behaviour with changes in age or the environment, further research is needed to explore how sex differences in the existence of activity‐aggression behavioural syndromes change across contexts. Furthermore, understanding the genetic underpinning of sex differences in a behavioural syndrome would be pivotal to assess the role of behavioural syndromes in the evolution of sexual dimorphism in behaviours.

## Graphical abstract

Males and females of the same species may not only differ in average behaviour but also differ in correlated suites of behaviours. We found that more active male field crickets were more aggressive towards other males. However, this behavioural correlation was not observed in females. Understanding sex‐specific behavioural correlations is important for predicting the evolution and maintenance of sex differences in behaviours.

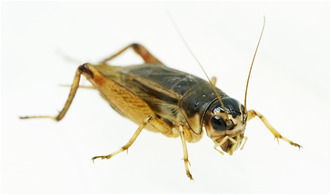

## 1 INTRODUCTION

Individual‐level behavioural studies have reported that individuals differ in their average behaviour, a phenomenon called animal personality, in many animal taxa (Dall & Griffith, 2014; Dingemanse, Kazem, Réale, & Wright, 2010; Réale et al., 2007). Moreover, repeatable components of different types of behaviour may be associated; this association is referred to as behavioural syndromes or among‐individual behavioural correlations (Dingemanse et al., 2012; Dingemanse, Dochtermann, & Wright, 2010). Such behavioural syndromes can constrain the independent evolution of correlated behaviours (Dochtermann & Dingemanse, 2013). For example, field crickets (*Gryllus integer*) expressed different average levels of exploration and boldness between populations but exhibited a consistent direction of the exploration‐boldness behavioural syndrome (Royauté et al., 2020). Considering that interactions among multiple behavioural traits can influence how a single behaviour is shaped by selection, the evaluation of behavioural syndromes is important for predicting behavioural responses to selection.

Behavioural syndromes can also differ between males and females within a species. Because males and females of the same species share most of their genome, genetic correlations between males and females (i.e. cross‐sex genetic correlations, *r*_MF_) in the same trait are not different from 1 (i.e. *r*_MF_ ≈ 1, Poissant et al., 2010), and behavioural syndromes are expected to be similar between sexes. However, when sex‐specific phenotypic optima are favoured, the strength or direction of selection differs between sexes (Bonduriansky & Chenoweth, 2009; Cox & Calsbeek, 2009; Singh & Punzalan, 2018). In particular, when each sex gains a fitness advantage from different associations among behavioural traits (i.e. sex‐specific correlational selection, Sinervo & Svensson, 2002), sex‐specific behavioural syndromes can arise. In other words, selection can favour sex‐specific magnitude or direction of behavioural syndromes. Moreover, when weak cross‐sex genetic correlations occur in multiple behavioural traits within a syndrome, genetic decoupling between sexes in those behavioural traits can result in sex‐specific directions or strengths of behavioural syndromes. Weak cross‐sex genetic correlations (i.e. *r*_MF_ ≈ 0) can facilitate the genetic decoupling of homologous traits between males and females (Chenoweth & McGuigan, 2010; Lande, 1980) and thereby generate sex differences in homologous traits between the sexes. As a result, a trait can be independently expressed in each sex and become sexually dimorphic. Therefore, as the sex specificity of behavioural syndromes can reflect past sex‐specific selection on behaviours or imply current genetic decoupling of behaviours between sexes, investigating sex‐specific behavioural syndromes is important for predicting behavioural evolution in males and females.

Despite this possibility, few studies have tested sex‐specific behavioural syndromes (Fresneau et al., 2014; Han et al., 2015; Michelangeli et al., 2016; Royauté et al., 2015; Way et al., 2015). Most of these studies did not rigorously test sex‐specificity in behavioural syndromes using a robust experimental design and rigorous statistical analysis. For example, some previous research showing sex‐specific behavioural syndromes did not use a repeated‐measures design but collected a single measurement of behaviour per individual (e.g. Han et al., 2015; Way et al., 2015). These studies simply calculated behavioural correlations at the phenotypic level and compared the correlation structures between sexes. However, to calculate behavioural syndromes and compare them between sexes, repeated individual‐level measures of behaviours are essential to decompose phenotypic behavioural correlations into within‐ and among‐individual behavioural correlations (Dingemanse & Dochtermann, 2013; Dingemanse & Wright, 2020). However, even among studies that measured behaviours repeatedly at the individual level, some did not use multivariate mixed‐effects models to distinguish between among‐individual and within‐individual behavioural correlations (e.g. Michelangeli et al., 2016); others did not explicitly test sex differences in behavioural syndromes but showed only differences in the signs of behavioural syndromes (e.g. Fresneau et al., 2014). For example, Fresneau et al. (2014) studied blue tits (*Cyanistes caeruleus*) and showed that males and females differed in the signs of behavioural syndromes. Females exhibited a negative among‐individual correlation between handling aggression and nestling defence, whereas males had a positive correlation that did not differ from zero (Fresneau et al., 2014). However, it was unclear whether the signs of the behavioural syndrome significantly differed between sexes. Therefore, to date, no empirical studies with a robust experimental design and rigorous statistical analysis have demonstrated sex differences in behavioural syndromes.

**FIGURE 1:**
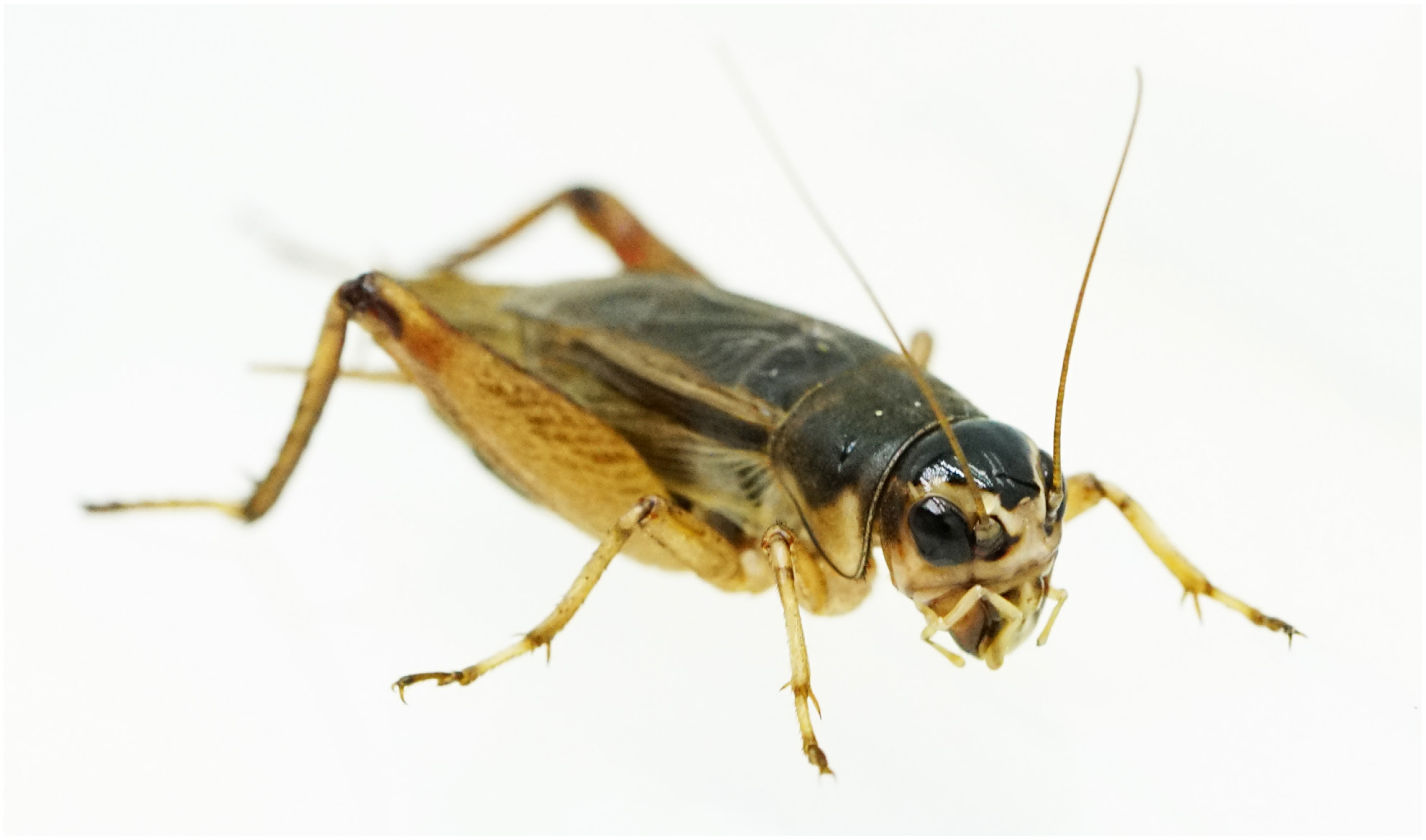
A field cricket *Teleogryllus emma*. Image credit: Chang Seok Han.

In this study, we assessed the sex specificity of behavioural syndromes in the field cricket *Teleogryllus emma* (Figure 1). Although its life history and taxonomy have previously been documented (Kim et al., 2007, 2008; Lu et al., 2018), the behaviour of this species remains relatively unexplored (but see Honda‐Sumi, 2005). However, based on our observations, the behaviour of *T. emma* is very similar to those of the field cricket *Gryllus bimaculatus* (C.S. Han, personal observation), which has been intensively studied as a model species in the fields of evolutionary biology and behavioural ecology (e.g. Adamo & Hoy, 1995; Bateman et al., 2001; Bretman et al., 2004; Rantala & Roff, 2005; Rantala & Kortet, 2003; Simmons, 1986a, 1986b, 1987, 1988, 1990; Tregenza & Wedell, 1998 and many other studies). During their reproductive period, *T. emma* males attract females with songs produced from within burrows or beneath vegetation (C.S. Han, personal observation). Females, in response, approach and mate with the preferred male (C.S. Han, personal observation). Males also respond to other males aggressively and express escalating aggressive behaviours (e.g. antennal fencing, mandible flare, mandible engagement, physical combat) when the opponent does not retreat (C.S. Han, personal observation). Given the behavioural similarities between *G. bimaculatus* and *T. emma*, we expected to find a positive activity‐aggression behavioural syndrome in *T. emma*, similar to that found in *G. bimaculatus* (Han et al., 2019; Santostefano et al., 2016) and other field cricket species (Kortet & Hedrick, 2007). Furthermore, if males and females substantially differed in their activity and aggression, sex‐specific selection on these behaviours could contribute to sex differences in behavioural syndromes. To address these questions, following the protocol of behavioural assays for *G. bimaculatus* (Han & Dingemanse, 2017b), we repeatedly measured the activity and aggression of *T. emma* males and females at the individual level, subsequently employing mixed‐effects models to estimate behavioural syndromes in males and females.

## 2 METHODS AND MATERIALS

### 2.1 Study species and rearing conditions

*Teleogryllus emma* is a species of field cricket widely distributed in East Asia. In Korea, *T. emma* has a univoltine life cycle in which overwintered eggs hatch in early summer, nymphs develop until September, and adults reproduce and lay eggs in September and October. We collected field crickets from the wild population near Jiri Mountain, Korea, in October 2017. Crickets were transported to a climate‐controlled room and housed individually in plastic rearing containers (diameter: 10 cm, height: 10 cm). Individuals were marked on the pronotum with enamel paint. Each container was provided with shelter (an egg carton) and ad libitum food (dog food) and water, which we replaced every 3 days. All individuals were housed at 26°C with 30%–50% relative humidity under a 12 L:12 D photoperiod.

### 2.2 Assays

Forty‐four males and 54 females were acclimated to the rearing containers for 1 week and then subjected to a set of assays to measure two behaviours at the individual level: activity and aggression. Each individual was assessed on each of two behaviours four times with a 2‐day interval. Each individual's body mass was repeatedly measured to the nearest 0.001 g at the end of the behavioural assays. After completing a set of behavioural assays, adults were returned to their home containers.

Activity—We removed individuals from the rearing containers and placed them in a plastic arena with a removable opaque partition in the middle, creating two smaller compartments (15 × 15 × 20 cm). A thin rubber mat was placed on the bottom of the arena to facilitate cricket movement. We measured each individual's total distance moved in the compartment for 15 min with the tracking software Noldus Ethovision XT 14 (Noldus Information Technology).

Aggression—Prior to the activity assay, we randomly assigned same‐sex individuals into dyads and placed each individual in individual compartments of the same arena for the activity assay. After the activity assay, we removed the partition in the middle of the arena and allowed the two same‐sex individuals to interact. Such assays based on dyadic interactions between same‐sex individuals have been widely used in the aggression assays in field crickets (Abbey‐Lee et al., 2018; DiRienzo et al., 2012; Han et al., 2018, 2019; Han & Dingemanse, 2017a; Kortet & Hedrick, 2007; Niemelä et al., 2012; Santostefano et al., 2016). In each trial in the aggression assay, one individual was randomly assigned as the focal individual and the other was labelled the partner. During each of the repeated aggression assays, individuals were assigned different partners (i.e. the same dyad did not reoccur). When *T. emma* males encounter each other, they typically show a hierarchy of aggressive behaviours, progressing from low‐intensity aggression (e.g. antennal fencing) to high‐intensity aggression (e.g. flaring mandibles, aggressive song stridulation and biting; C.S. Han, personal observation). An aggressive encounter (contest) was considered to end when one individual retreated. Even after the first fight ended, with a clear winner and loser, the winner male sometimes continued to chase the loser male and exhibit aggressive behaviours, whereas the loser male rarely exhibited aggressive behaviours. Thus, we defined individual aggression as the duration of the first fight (in seconds, from the initiation of the first aggressive behaviour to the retreat of one individual). Each aggression assay lasted 15 minutes.

### 2.3 Statistical analyses

First, to evaluate average phenotypic differences between sexes, we constructed univariate mixed‐effects models where each z‐transformed trait (activity, aggression and body mass) was fitted as a response variable. Behavioural traits were log‐transformed (ensuring normally distributed residuals according to visual inspection) prior to the *z*‐transformation. In the univariate models, we included sex (two‐level factor: male vs. female), testing order (the order of the repeated measurements, covariate) and their interaction as fixed factors and individual identity as a random factor. In the aggression analysis, interaction partner identity was also included in the model as an additional random factor to estimate how much phenotypic variation in aggression was explained by partner identity. The significance of fixed effects was determined using conditional Wald F tests. The significance of random effects was derived from likelihood ratio tests (LRTs) calculated as the *χ* ^2^‐distributed difference in deviance (−2 × log likelihood) between the full model and a model where the focal random effect was removed. The *p* value of variance components was calculated assuming an equal mixture of *p* (*χ* ^2^, df = 0) and *p* (*χ* ^2^, df = 1; Pinheiro & Bates, 2006; Self & Liang, 1987; Visscher, 2006), denoted as ‘$\chi_{0/1}^{2}$’ in the tables.

In addition, to estimate sex differences in among‐individual variances in the trait, we constructed distinct trait‐specific bivariate mixed‐effects models where each trait (log‐transformed activity and body mass) for each sex was fitted as two response variables per trait. A bivariate model for aggression was not built due to the absence of female aggression (see Section 3). In these bivariate models, we did not use *z*‐transformed variables but used log‐transformed (e.g. activity) or raw values (e.g. body mass) as response variables for comparing the extent of among‐individual trait variances between males and females. In the models, we did not include fixed factors and included individual identity as a random factor. As the bivariate model employed homologous traits of males and females as response variables, estimations of cross‐sex among‐ and within‐individual trait covariances were unfeasible and consequently set at zero. To assess sex differences in the extent of among‐individual variance for each trait, LRTs were calculated as the difference between a model where the among‐individual variance was separately estimated for each sex and a model where it was constrained to be the same between sexes, assuming one degree of freedom ($\chi_{1}^{2}$ in Tables). In addition to the comparison of among‐individual variances between males and females, we also calculated the sex‐specific repeatability, mean‐standardised among‐individual variance (Dochtermann & Royauté, 2019) and coefficient of among‐individual variances (Royauté & Dochtermann, 2021) and compared mean‐standardised among‐individual variances between sexes (see Table S1). In the analysis, we used the same structures of bivariate mixed‐effects models to test sex differences in mean‐standardised among‐individual variances in the trait. In those models, mean‐standardised trait values (a trait value divided by sex‐specific average trait value) were fitted as response variables.

Finally, to test whether males and females differed in the sign of among‐individual correlations between traits, we combined the trait‐specific bivariate mixed‐effect models above into a single multivariate mixed‐effects model. Because females did not express any aggressive behaviour, we tested the sex difference only in the among‐individual correlation between activity and body mass. In this model, we did not include fixed factors and included individual identity as a random factor. Cross‐sex trait covariances were constrained to zero at both among‐individual and within‐individual levels, whereas within‐sex trait covariances were allowed to vary freely. To test whether a within‐sex among‐individual trait correlation was larger than 0, LRTs were calculated as the difference between a model where the correlation was estimated or removed, assuming one degree of freedom ($\chi_{1}^{2}$ in Tables). To assess the sex differences in among‐individual correlations, the LRTs were calculated as the difference in deviance between a model where among‐individual correlations were separately estimated for each sex and one in which among‐individual correlations were constrained to be the same between sexes, assuming one degree of freedom ($\chi_{1}^{2}$ in Tables).

All models above were implemented in ASReml (ver. 4.1; VSN Interaction) and solved using the restricted maximum likelihood.

## 3 RESULTS

Males and females did not differ in activity (Table 1). However, males were more aggressive and had a lower body mass than females (Table 1). Females did not express any aggressive behaviours toward other females. Instead, females occasionally approached the other individual and remained in close proximity without engaging in specific behaviour such as antenna touching. Females gained weight over time during the repeated assays, but males did not (Table 1). Male and female behaviours did not change over the repeated assays (Table 1).

**TABLE 1:** Linear mixed‐effects model of aggression, activity and body mass as a function of sex, testing order and their interaction.

Fixed effects	Aggression			Activity			Body mass		
	*β* (SE)	*F*_NUMdf,DENdf_	*p*	*β* (SE)	*F*_NUMdf,DENdf_	*p*	*β* (SE)	*F*_NUMdf,DENdf_	*p*
Intercept	−0.82 (0.12)	0.72_1,138.0_	.40	−0.01 (0.16)	2.11_1,390.1_	.15	0.73 (0.07)	0.03_1,125.8_	.85
Testing order	0.00 (0.04)	1.04_1,102.4_	.31	0.01 (0.06)	2.81_1,299.1_	.10	0.05 (0.01)	0.18_1,293.1_	.67
Sex^a^	1.65 (0.17)	371.05_1,80.6_	<.001	−0.32 (0.23)	0.10_1,101.6_	.75	−1.48 (0.10)	275.77_1,105.7_	<.001
Testing order × sex	0.06 (0.06)	1.26_1,102.2_	.26	0.12 (0.08)	2.10_1,299.2_	.15	−0.09 (0.01)	43.32_1,293.1_	<.001

Random effects	*σ* ^2^ (SE)	$\chi_{0/1}^{2}$	*p*	*σ* ^2^ (SE)	$\chi_{0/1}^{2}$	*p*	*σ* ^2^ (SE)	$\chi_{0/1}^{2}$	*p*
Focal ID	0.11 (0.03)	23.70	<.001	0.23 (0.06)	22.04	<.001	0.27 (0.04)	632.06	<.001
Partner ID	0.00 (−)	0.00	.50						
Residual	0.11 (0.02)			0.77 (0.06)			0.02 (0.002)		

*Note*: Parameters are provided with standard errors in parentheses.

^a^ Reference category: female.

In both males and females, individuals varied in their activity and body mass (Table 2). The extent of among‐individual variance in activity did not differ between males and females ($\chi_{1}^{2}$ = 2.22, *p* = .14), whereas the extent of among‐individual variance in body mass was significantly greater in females than in males ($\chi_{1}^{2}$ = 11.22, *p* < .001). However, the mean‐standardised among‐individual variances in activity and body mass did not differ between sexes (activity: $\chi_{1}^{2}$ = 0.08, *p* = .77; body mass: $\chi_{1}^{2}$ = 2.29, *p* = .13; Table S1). Male aggression also differed among individuals (Table 2), but was not affected by partner identity (Table 1). As females did not express any aggressive behaviours toward other females, the among‐individual variance in female aggression was zero. Instead, as we found that females showed proximity‐seeking behaviour rather than aggression, we analysed the duration in which the focal female was in close proximity to the female interacting partner during the aggression assay and examined whether there was any significant among‐individual variation in this behaviour. However, our analysis revealed that this behaviour was not repeatable at the individual level (see Text S1).

**TABLE 2:** Among‐individual variances in log‐transformed aggression, log‐transformed activity and body mass in males and females with standard errors in parentheses.

	Males			Females		
	*σ* ^2^ (SE)	$\chi_{0/1}^{2}$	*p*	*σ* ^2^ (SE)	$\chi_{0/1}^{2}$	*p*
Aggression	0.70 (0.27)	11.25	<.001	NA	NA	NA
Activity	0.15 (0.05)	12.26	<.001	0.06 (0.03)	4.12	.02
Body mass	1.25 × 10^−2^ (0.26 × 10^−2^)	195.12	<.001	3.29 × 10^−2^ (0.65 × 10^−2^)	364.40	<.001

*Note*: The significance of among‐individual variance was determined with a likelihood‐ratio test comparing the focal model with and without the variance (see Section 2).

**FIGURE 2:**
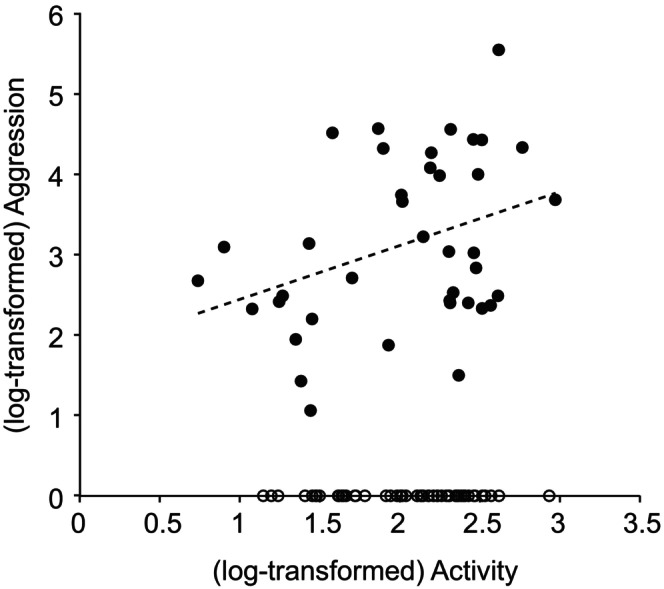
The activity‐aggression behavioural syndrome in male (closed circles) and female (open circles) *Teleogryllus emma*. Each dot indicates the mean value of each individual's log‐transformed behaviour.

The among‐individual correlation (behavioural syndrome) between activity and aggression could not be defined in females due to the absence of the among‐individual variance in female aggression (Table 3a, Figure 2). However, behavioural syndrome between activity and aggression was significantly greater than zero in males; in other words, more active males were more aggressive (Table 3a, Figure 2).

**TABLE 3:** (a) Among‐ and (b) within‐individual trait correlations in males and females with standard errors in parentheses.

(a) Among‐individual trait correlations (*r*_*i*_s)						
	Males			Females		
	*r*_*i*_ (SE)	Test *r* = 0, $\chi_{1}^{2}$	*p*	*r*_*i*_ (SE)	Test *r* = 0, $\chi_{1}^{2}$	*p*
Fight duration—Activity	0.65 (0.25)	6.48	.01	NA	NA	NA
Fight duration—Body mass	−0.02 (0.20)	0.01	.93	NA	NA	NA
Activity—Body mass	0.27 (0.18)	2.36	.12	0.09 (0.22)	0.17	.68

(b) Within‐individual trait correlations (*r*_*w*_s)						
	Males			Females		
	*r*_*w*_ (SE)	Test *r* = 0, $\chi_{1}^{2}$	*p*	*r*_*w*_ (SE)	Test *r* = 0, $\chi_{1}^{2}$	*p*
Fight duration—Activity	−0.13 (0.15)	0.73	.39	NA	NA	NA
Fight duration—Body mass	−0.16 (0.17)	0.63	.42	NA	NA	NA
Activity—Body mass	−0.04 (0.05)	0.30	.44	−0.15 (0.08)	3.40	.07

*Note*: The significance was based on chi‐squared values derived from likelihood‐ratio tests comparing the models described in Section 2.

The among‐individual correlations between activity and body mass were not different from zero in males or females (Table 3a), and the strengths of these correlations did not differ between males and females ($\chi_{1}^{2}$ = 0.46, *p* = .50). Within‐individual correlations, between traits were not different from zero in males or females (Table 3b).

## 4 DISCUSSION

*Teleogryllus emma* males displayed a strong positive behavioural syndrome between activity and aggression. More active and aggressive males may collect and retain more resources, attract more females and accrue more mating opportunities during their short reproductive period (duration of 4–8 weeks) than less active and aggressive males. Survival and reproductive advantages for more active and aggressive males have been found in field cricket species. For example, female field crickets (*Gryllus assimilis*) prefer mating with dominant males rather than with subordinate males (Loranger & Bertram, 2016). A positive correlation between aggression and signalling efforts in *G. assimilis* males also suggests that females are more likely to mate with more aggressive males (Bertram & Rook, 2012). In the field cricket *G. bimaculatus*, the survival rate of more active and more aggressive males was greater (Han & Dingemanse, 2017a). Consequently, selection is expected to favour males who are both more active and more aggressive, leading to a positive behavioural syndrome between activity and aggression in males. In multiple field cricket species, dominant male crickets were more exploratory and active than subordinate males (e.g., *Teleogryllus oceanicus*, Rudin et al., 2017; *Gryllus integer*, Kortet & Hedrick, 2007), indicating that activity‐aggression behavioural syndrome might be widespread in male field crickets.

However, the activity‐aggression behavioural syndrome was absent in *T. emma* females because females do not engage in aggressive fights. No instances of aggression were observed in the dyadic tests in *T. emma* females, indicating a lack of aggression in female adults of this species. Although our aggression assay, which involved measuring individual aggressive behaviour during dyadic interactions between same‐sex individuals, has been widely used for assessing aggression in field crickets (Abbey‐Lee et al., 2018; DiRienzo et al., 2012; Han et al., 2018, 2019; Han & Dingemanse, 2017a; Kortet & Hedrick, 2007; Niemelä et al., 2012; Santostefano et al., 2016), the absence of aggression in *T. emma* females might be attributed to the specific conditions of our aggression assay. For example, female field crickets, *Gryllus campestris*, become aggressive toward other females only in the presence of nearby calling males (Rillich et al., 2009). They are also known to attack heterospecific males to avoid mating but not conspecific males (Tyler et al., 2015). If the aggression of female field crickets *T. emma* is also highly plastic and context specific, we may need to consider using different assay conditions (adding additional males or using heterospecific males rather than conspecific females) to measure female aggression.

The absence of aggression in *T. emma* females in our study might also be due to the ad libitum food provided in the laboratory. Nutritional conditions play a crucial role in determining the extent of among‐individual variation in behaviour (Han et al., 2019; Han & Dingemanse, 2017a, 2017b; Kelleher et al., 2019; Royauté et al., 2019), and thus, the presence of among‐individual correlations among traits is expected to be influenced by nutritional conditions (Han et al., 2019). In contrast to the laboratory environment where ad libitum food is provided, female field crickets in their natural habitat, with limited access to food resources, may exhibit aggression and show variation in aggression among individuals. For example, female crickets *Acheta domesticus* were found to rarely interact with other females in the laboratory but engaged in aggressive fights over food when starved (Nosil, 2002). Therefore, as we conducted behavioural assays on wild‐caught individuals that had been reared for 1 week with unrestricted access to food, the lack of aggression in *T. emma* females could be attributed to the resource‐rich laboratory environment. However, a quantitative genetics study on the field cricket *G. bimaculatus* revealed that genetic associations between behavioural and morphological traits were stronger in both sexes under high‐quality (free‐choice) diets than under low‐quality (protein‐deprived) diets (Han et al., 2019). These results suggest that behavioural syndromes could be more pronounced under favourable nutritional conditions, which is the opposite of the above prediction of changes in behavioural syndromes between resource‐poor wild environments and resource‐rich laboratory environments.

In addition, the absence of aggression in *T. emma* females might be due to the age of the female individuals collected from the wild. Older adult field crickets are expected to exhibit increased risk‐taking and aggression compared to younger adults, influenced by the decrease in future reproductive prospects with age (‘asset protection hypothesis’, Clark, 1994). This suggests the potential for aggression and the emergence of activity‐aggression behavioural syndromes among older adult *T. emma* females. Nevertheless, considering that our collection of *T. emma* individuals took place during their reproductive peak in the field, it appears improbable that the absence of aggression in all adult females can be attributed to age. Instead, female *T. emma* nymphs might exhibit greater aggression than adults. It is plausible that *T. emma* nymphs, especially female nymphs, have higher protein and food resource requirements than adults, as observed in *G. bimaculatus* (Han & Dingemanse, 2017b). Therefore, female *T. emma* nymphs might behave aggressively to consume more resources such as protein, potentially leading to the presence of behavioural syndromes linking activity and aggression in female nymphs.

Considering that female aggression in field crickets is highly plastic and context specific, sex differences in the existence of the activity‐aggression behavioural syndrome in *T. emma* might depend on the context. Alternatively, *T. emma* females may not engage in aggressive fights regardless of context because of the lack of selection favouring female aggression in this species. Previous research on aggression in the field cricket *G. bimaculatus* has shown higher aggression levels in males than in females (Han & Dingemanse, 2017a, 2017b), and the strength of directional selection for aggression tended to be higher in males than in females (Han & Dingemanse, 2017a). Thus, despite the possibility that aggression in different contexts is positively associated with activity in *T. emma* females, the lack of selection favouring aggression in *T. emma* females across contexts may have led to the absence of aggression and related behavioural syndromes in females.

Sex differences in behavioural syndromes suggest that the genetic correlations among behaviours may vary between sexes, potentially leading to greater disparities in behaviours between sexes in the future. Genetic correlations underlying behavioural syndromes can restrict the independent evolution of correlated traits (Dochtermann & Dingemanse, 2013). When two behaviours are strongly genetically linked, evolutionary changes in one behaviour can influence the other. Consequently, when behavioural genetic correlations are conserved, the signs of behavioural syndromes can be similar across populations in different environments. For example, populations of field crickets (*G. integer*) exhibited variations in average behaviours (exploration and boldness), but their behavioural syndrome structures remained consistent due to the conserved behavioural genetic correlations (Royauté et al., 2020). Similarly, sex differences in behavioural syndromes are expected to shape sex‐specific evolutionary responses and ultimately contribute to greater sexual dimorphism in behaviours. In *T. emma*, males exhibited a positive activity‐aggression syndrome, indicating that the evolution of these two behaviours is likely to occur rapidly along the ‘genetic line of least genetic resistance’ (Schluter, 1996). In contrast, *T. emma* females did not exhibit an activity‐aggression syndrome, suggesting that the evolution of these two behaviours in response to selection will be independent. In the other words, sex differences in the existence of behavioural syndromes can either enhance or maintain sex differences in behaviours.

In our study, we demonstrated activity‐aggression behavioural syndromes in males but not in females of the field cricket *T. emma*. Sex differences in the existence of behavioural syndromes may play a pivotal role in the evolution of sex differences in behaviours by reflecting the sex‐specific genetic correlation of these behaviours. However, the discrepancy between behavioural syndromes and the genetic correlations among behaviours can be greater, as the contribution of the (permanent) environmental correlations to among‐individual correlations is not negligible (e.g. Petelle et al., 2015). For instance, in a study of Trinidadian guppies (*Poecilia reticulata*), the variance–covariance matrices of behaviours did not differ between males and females at the individual or genotype levels (White et al., 2019). However, in yellow‐bellied marmots (*Marmota flaviventris*), the permanent environmental correlation between docility and exploration was significantly positive, while the genetic correlation between these behaviours was not (Petelle et al., 2015). Consequently, a significant positive behavioural syndrome emerged in the absence of a genetic correlation. Therefore, it is crucial to identify sex differences in the genetic basis of behavioural syndromes to comprehend the role of behavioural syndromes in the evolution of sex differences in behaviour. Moreover, as behavioural syndromes may be linked to other types of traits (e.g. life‐history, physiological, or morphological traits), future studies should strive to assess the differences in individual‐ and genetic‐level associations among multiple types of traits between males and females (Hämäläinen et al., 2018) and investigate how these distinctions contribute to the evolution of sexual dimorphism.

## AUTHOR CONTRIBUTIONS

**Chang S. Han:** Conceptualization (equal); data curation (equal); formal analysis (equal); funding acquisition (equal); investigation (equal); writing – original draft (equal); writing – review and editing (equal). **Byeongho Lee:** Data curation (equal); formal analysis (equal); investigation (equal). **Jong‐yeol Moon:** Data curation (equal).

## CONFLICT OF INTEREST STATEMENT

We declare that we have no competing interests.

## Supporting information


Data S1

## Data Availability

All data are available at https://shorturl.at/hpxCL.

## References

[ece310642-bib-0001] Abbey‐Lee, R. N. , Uhrig, E. J. , Garnham, L. , Lundgren, K. , Child, S. , & Løvlie, H. (2018). Experimental manipulation of monoamine levels alters personality in crickets. Scientific Reports, 8(1), 16211. 30385805 10.1038/s41598-018-34519-z PMC6212410

[ece310642-bib-0002] Adamo, S. A. , & Hoy, R. R. (1995). Agonistic behaviour in male and female field crickets, *Gryllus bimaculatus*, and how behavioural context influences its expression. Animal Behaviour, 49(6), 1491–1501.

[ece310642-bib-0003] Bateman, P. W. , Gilson, L. N. , & Ferguson, J. (2001). Male size and sequential mate preference in the cricket *Gryllus bimaculatus* . Animal Behaviour, 61(3), 631–637.

[ece310642-bib-0004] Bertram, S. M. , & Rook, V. (2012). Relationship between condition, aggression, signaling, courtship, and egg laying in the field cricket, *Gryllus assimilis* . Ethology, 118(4), 360–372.

[ece310642-bib-0005] Bonduriansky, R. , & Chenoweth, S. F. (2009). Intralocus sexual conflict. Trends in Ecology & Evolution, 24(5), 280–288. 19307043 10.1016/j.tree.2008.12.005

[ece310642-bib-0006] Bretman, A. , Wedell, N. , & Tregenza, T. (2004). Molecular evidence of post–copulatory inbreeding avoidance in the field cricket *Gryllus bimaculatus* . Proceedings of the Royal Society of London. Series B: Biological Sciences, 271(1535), 159–164. 10.1098/rspb.2003.2563 PMC1691572 15058392

[ece310642-bib-0007] Chenoweth, S. F. , & McGuigan, K. (2010). The genetic basis of sexually selected variation. Annual Review of Ecology, Evolution, and Systematics, 41, 81–101.

[ece310642-bib-0008] Clark, C. W. (1994). Antipredator behavior and the asset‐protection principle. Behavioral Ecology, 5(2), 159–170.

[ece310642-bib-0009] Cox, R. M. , & Calsbeek, R. (2009). Sexually antagonistic selection, sexual dimorphism, and the resolution of intralocus sexual conflict. The American Naturalist, 173(2), 176–187. 10.1086/595841 19138156

[ece310642-bib-0010] Dall, S. R. , & Griffith, S. C. (2014). An empiricist guide to animal personality variation in ecology and evolution. Frontiers in Ecology and Evolution, 2, 3.

[ece310642-bib-0011] Dingemanse, N. , Dochtermann, N. , & Wright, J. (2010). A method for exploring the structure of behavioural syndromes to allow formal comparison within and between data sets. Animal Behaviour, 79(2), 439–450.

[ece310642-bib-0012] Dingemanse, N. J. , & Dochtermann, N. A. (2013). Quantifying individual variation in behaviour: Mixed‐effect modelling approaches. Journal of Animal Ecology, 82(1), 39–54. 23171297 10.1111/1365-2656.12013

[ece310642-bib-0013] Dingemanse, N. J. , Dochtermann, N. A. , & Nakagawa, S. (2012). Defining behavioural syndromes and the role of ‘syndrome deviation’ in understanding their evolution. Behavioral Ecology and Sociobiology, 66(11), 1543–1548.

[ece310642-bib-0014] Dingemanse, N. J. , Kazem, A. J. N. , Réale, D. , & Wright, J. (2010). Behavioural reaction norms: Animal personality meets individual plasticity. Trends in Ecology & Evolution, 25(2), 81–89. 19748700 10.1016/j.tree.2009.07.013

[ece310642-bib-0015] Dingemanse, N. J. , & Wright, J. (2020). Criteria for acceptable studies of animal personality and behavioural syndromes. Ethology, 126(9), 865–869.

[ece310642-bib-0016] DiRienzo, N. , Pruitt, J. N. , & Hedrick, A. V. (2012). Juvenile exposure to acoustic sexual signals from conspecifics alters growth trajectory and an adult personality trait. Animal Behaviour, 84(4), 861–868.

[ece310642-bib-0017] Dochtermann, N. A. , & Dingemanse, N. J. (2013). Behavioral syndromes as evolutionary constraints. Behavioral Ecology, 24(4), 806–811.

[ece310642-bib-0018] Dochtermann, N. A. , & Royauté, R. (2019). The mean matters: Going beyond repeatability to interpret behavioural variation. Animal Behaviour, 153, 147–150.

[ece310642-bib-0019] Fresneau, N. , Kluen, E. , & Brommer, J. E. (2014). A sex‐specific behavioral syndrome in a wild passerine. Behavioral Ecology, 25(2), 359–367.

[ece310642-bib-0020] Hämäläinen, A. , Immonen, E. , Tarka, M. , & Schuett, W. (2018). Evolution of sex‐specific pace‐of‐life syndromes: Causes and consequences. Behavioral Ecology and Sociobiology, 72(3), 1–15. 10.1007/s00265-018-2462-1 PMC5856903 29576676

[ece310642-bib-0021] Han, C. S. , & Dingemanse, N. J. (2017a). Sex‐dependent expression of behavioural genetic architectures and the evolution of sexual dimorphism. Proceedings of the Royal Society B: Biological Sciences, 284(1864), 20171658. 10.1098/rspb.2017.1658 PMC5647308 28978735

[ece310642-bib-0022] Han, C. S. , & Dingemanse, N. J. (2017b). You are what you eat: Diet shapes body component, personality and behavioural stability. BMC Evolutionary Biology, 17, 8. 28073352 10.1186/s12862-016-0852-4 PMC5223362

[ece310642-bib-0023] Han, C. S. , Gosden, T. P. , & Dingemanse, N. J. (2019). Protein deprivation facilitates independent evolution of behaviour and morphology. Evolution, 73, 1809–1820. 31318455 10.1111/evo.13802

[ece310642-bib-0024] Han, C. S. , Jablonski, P. G. , & Brooks, R. C. (2015). Intimidating courtship and sex differences in predation risk lead to sex‐specific behavioural syndromes. Animal Behaviour, 109, 177–185.

[ece310642-bib-0025] Han, C. S. , Tuni, C. , Ulcik, J. , & Dingemanse, N. J. (2018). Increased developmental density decreases the magnitude of indirect genetic effects expressed during agonistic interactions in an insect. Evolution, 72, 2435–2448. 30221347 10.1111/evo.13600

[ece310642-bib-0026] Honda‐Sumi, E. (2005). Difference in calling song of three field crickets of the genus *Teleogryllus*: The role in premating isolation. Animal Behaviour, 69(4), 881–889.

[ece310642-bib-0027] Kelleher, S. R. , Silla, A. J. , Niemelä, P. T. , Dingemanse, N. J. , & Byrne, P. G. (2019). Dietary carotenoids affect the development of individual differences and behavioral plasticity. Behavioral Ecology, 30(5), 1273–1282.

[ece310642-bib-0028] Kim, N. , Hong, S. J. , Seol, K. Y. , & Kim, S. H. (2008). Short daylengths accelerate nymphal development of the emma field cricket, *Teleogryllus emma* (Orthoptera: Gryllidae). Journal of Asia‐Pacific Entomology, 11(1), 13–15.

[ece310642-bib-0029] Kim, N.‐J. , Hong, S.‐J. , Seol, K.‐Y. , Kim, S.‐H. , Ahn, N.‐H. , & Kim, M. (2007). Effect of temperature on development and reproduction of the emma field cricket, *Teleogryllus emma* (Orthoptera: Gryllidae). International Journal of Industrial Entomology, 15(1), 69–73.

[ece310642-bib-0030] Kortet, R. , & Hedrick, A. (2007). A behavioural syndrome in the field cricket *Gryllus integer*: Intrasexual aggression is correlated with activity in a novel environment. Biological Journal of the Linnean Society, 91(3), 475–482.

[ece310642-bib-0032] Lande, R. (1980). Sexual dimorphism, sexual selection, and adaptation in polygenic characters. Evolution, 34, 292–305. 28563426 10.1111/j.1558-5646.1980.tb04817.x

[ece310642-bib-0033] Loranger, M. J. , & Bertram, S. M. (2016). The effect of male dominance on female choice in a field cricket (*Gryllus assimilis*). Animal Behaviour, 114, 45–52.

[ece310642-bib-0034] Lu, H. , Wang, X.‐Y. , Wang, H.‐Q. , Li, K. , & He, Z.‐Q. (2018). A taxonomic study of genus teleogryllus from East Asia (Insecta: Orthoptera: Gryllidae). Journal of Asia‐Pacific Entomology, 21(2), 667–675.

[ece310642-bib-0035] Michelangeli, M. , Chapple, D. G. , & Wong, B. (2016). Are behavioural syndromes sex specific? Personality in a widespread lizard species. Behavioral Ecology and Sociobiology, 70(11), 1911–1919.

[ece310642-bib-0036] Niemelä, P. T. , Vainikka, A. , Lahdenperä, S. , & Kortet, R. (2012). Nymphal density, behavioral development, and life history in a field cricket. Behavioral Ecology and Sociobiology, 66(5), 645–652.

[ece310642-bib-0037] Nosil, P. (2002). Food fights in house crickets, *Acheta domesticus*, and the effects of body size and hunger level. Canadian Journal of Zoology, 80(3), 409–417.

[ece310642-bib-0038] Petelle, M. B. , Martin, J. G. , & Blumstein, D. T. (2015). Heritability and genetic correlations of personality traits in a wild population of yellow‐bellied marmots (*Marmota flaviventris*). Journal of Evolutionary Biology, 28(10), 1840–1848. 26214760 10.1111/jeb.12700

[ece310642-bib-0039] Pinheiro, J. , & Bates, D. (2006). Mixed‐effects models in S and S‐PLUS. Springer.

[ece310642-bib-0040] Poissant, J. , Wilson, A. J. , & Coltman, D. W. (2010). Sex‐specific genetic variance and the evolution of sexual dimorphism: A systematic review of cross‐sex genetic correlations. Evolution, 64(1), 97–107. 19659596 10.1111/j.1558-5646.2009.00793.x

[ece310642-bib-0041] Rantala, M. , & Roff, D. (2005). An analysis of trade‐offs in immune function, body size and development time in the Mediterranean field cricket, *Gryllus bimaculatus* . Functional Ecology, 19, 323–330.

[ece310642-bib-0042] Rantala, M. J. , & Kortet, R. (2003). Courtship song and immune function in the field cricket *Gryllus bimaculatus* . Biological Journal of the Linnean Society, 79(3), 503–510.

[ece310642-bib-0043] Réale, D. , Reader, S. M. , Sol, D. , McDougall, P. T. , & Dingemanse, N. J. (2007). Integrating animal temperament within ecology and evolution. Biological Reviews, 82(2), 291–318. 17437562 10.1111/j.1469-185X.2007.00010.x

[ece310642-bib-0044] Rillich, J. , Buhl, E. , Schildberger, K. , & Stevenson, P. A. (2009). Female crickets are driven to fight by the male courting and calling songs. Animal Behaviour, 77(3), 737–742.

[ece310642-bib-0045] Royauté, R. , Buddle, C. M. , & Vincent, C. (2015). Under the influence: Sublethal exposure to an insecticide affects personality expression in a jumping spider. Functional Ecology, 29(7), 962–970.

[ece310642-bib-0046] Royauté, R. , & Dochtermann, N. A. (2021). Comparing ecological and evolutionary variability within datasets. Behavioral Ecology and Sociobiology, 75(9), 127.

[ece310642-bib-0047] Royauté, R. , Garrison, C. , Dalos, J. , Berdal, M. A. , & Dochtermann, N. A. (2019). Current energy state interacts with the developmental environment to influence behavioural plasticity. Animal Behaviour, 148, 39–51.

[ece310642-bib-0048] Royauté, R. , Hedrick, A. , & Dochtermann, N. A. (2020). Behavioural syndromes shape evolutionary trajectories via conserved genetic architecture. Proceedings of the Royal Society B: Biological Sciences, 287(1927), 20200183. 10.1098/rspb.2020.0183 PMC7287377 32429805

[ece310642-bib-0049] Rudin, F. , Tomkins, J. , & Simmons, L. (2017). Changes in dominance status erode personality and behavioral syndromes. Behavioral Ecology, 28(1), 270–279.

[ece310642-bib-0050] Santostefano, F. , Wilson, A. J. , Araya‐Ajoy, Y. G. , & Dingemanse, N. J. (2016). Interacting with the enemy: Indirect effects of personality on conspecific aggression in crickets. Behavioral Ecology, 27, 1235–1246.

[ece310642-bib-0051] Schluter, D. (1996). Adaptive radiation along genetic lines of least resistance. Evolution, 50(5), 1766–1774. 28565589 10.1111/j.1558-5646.1996.tb03563.x

[ece310642-bib-0052] Self, S. G. , & Liang, K.‐Y. (1987). Asymptotic properties of maximum likelihood estimators and likelihood ratio tests under nonstandard conditions. Journal of the American Statistical Association, 82(398), 605–610.

[ece310642-bib-0053] Simmons, L. (1986a). Female choice in the field cricket *Gryllus bimaculatus* (De Geer). Animal Behaviour, 34(5), 1463–1470.

[ece310642-bib-0054] Simmons, L. (1986b). Inter‐male competition and mating success in the field cricket, *Gryllus bimaculatus* (De Geer). Animal Behaviour, 34(2), 567–579.

[ece310642-bib-0055] Simmons, L. (1987). Sperm competition as a mechanism of female choice in the field cricket, *Gryllus bimaculatus* . Behavioral Ecology and Sociobiology, 21(3), 197–202.

[ece310642-bib-0056] Simmons, L. (1988). The calling song of the field cricket, *Gryllus bimaculatus* (De Geer): Constraints on transmission and its role in intermale competition and female choice. Animal Behaviour, 36(2), 380–394.

[ece310642-bib-0057] Simmons, L. (1990). Post‐copulatory guarding, female choice and the levels of gregarine infections in the field cricket, *Gryllus bimaculatus* . Behavioral Ecology and Sociobiology, 26(6), 403–407.

[ece310642-bib-0058] Sinervo, B. , & Svensson, E. (2002). Correlational selection and the evolution of genomic architecture. Heredity, 89(5), 329–338. 12399990 10.1038/sj.hdy.6800148

[ece310642-bib-0059] Singh, A. , & Punzalan, D. (2018). The strength of sex‐specific selection in the wild. Evolution, 72(12), 2818–2824. 30298925 10.1111/evo.13625

[ece310642-bib-0060] Tregenza, T. , & Wedell, N. (1998). Benefits of multiple mates in the cricket *Gryllus bimaculatus* . Evolution, 52(6), 1726–1730. 28565303 10.1111/j.1558-5646.1998.tb02252.x

[ece310642-bib-0061] Tyler, F. , Fisher, D. , d'Ettorre, P. , Rodriguez‐Munoz, R. , & Tregenza, T. (2015). Chemical cues mediate species recognition in field crickets. Frontiers in Ecology and Evolution, 3, 48.

[ece310642-bib-0062] Visscher, P. M. (2006). A note on the asymptotic distribution of likelihood ratio tests to test variance components. Twin Research and Human Genetics, 9(4), 490–495. 16899155 10.1375/183242706778024928

[ece310642-bib-0063] Way, G. P. , Kiesel, A. L. , Ruhl, N. , Snekser, J. L. , & McRobert, S. P. (2015). Sex differences in a shoaling‐boldness behavioral syndrome, but no link with aggression. Behavioural Processes, 113, 7–12. 25562194 10.1016/j.beproc.2014.12.014

[ece310642-bib-0064] White, S. J. , Houslay, T. M. , & Wilson, A. J. (2019). Evolutionary genetics of personality in the Trinidadian guppy II: Sexual dimorphism and genotype‐by‐sex interactions. Heredity, 122(1), 15–28. 29795179 10.1038/s41437-018-0083-0 PMC6288163

