# Supplementary material for: Activity‐aggression behavioural syndromes exist in males but not in females of the field cricket Teleogryllus emma
Source: Ecol Evol. 2023 Oct 18;13(10):e10642. doi: 10.1002/ece3.10642 (PMC10582681; doi:10.1002/ece3.10642)
Supplement: Supplementary file 1 — Data S1 [file ECE3-13-e10642-s001.docx]

**Activity-aggression behavioural syndromes exist in males but not in females of the field cricket *Teleogryllus emma***

Chang S. Han ^a *^, Byeongho Lee ^a^ and Jong-yeol Moon ^a^

^a^ Department of Biology, Kyung Hee University, Seoul, Korea

**Supplementary Table S1.** The repeatability (*R*), mean-standardised among-individual variance (*I*, Dochtermann and Royaute 2019) and coefficient of among-individual variance (*CV_I_*, Royaute and Dochtermann 2021) of aggression, activity and body mass in males and females with standard errors in parentheses.

|  | Males | | |  | Females | | |
| --- | --- | --- | --- | --- | --- | --- | --- |
|  | *R* (SE) | *I* (SE) | *CV_I_* (SE) |  | *R* (SE) | *I* (SE) | *CV_I_* (SE) |
| Aggression | 0.49  (0.19) | 0.08  (0.03) | 0.28  (0.05) |  | NA | NA | NA |
| Activity | 0.27  (0.10) | 0.04  (0.01) | 0.19  (0.03) |  | 0.17  (0.08) | 0.02  (0.007) | 0.12  (0.03) |
| Body mass | 0.86  (0.18) | 0.02  (0.005) | 0.16  (0.02) |  | 0.96  (0.20) | 0.02  (0.005) | 0.15  (0.01) |

**Supplementary Text S1. Individual differences in the proximity-seeking behaviour of *Teleogryllus emma* females**

During the aggression assay, *Teleogryllus emma* females never attacked partner individuals. Instead, they occasionally remained in close proximity without exhibiting any specific behaviours. To assess variations in proximity-seeking among female individuals, we employed tracking software to measure the duration of time during which the focal individual was within 3 cm of the partner individual. We used a univariate mixed-effects model, fitting z-transformed log-transformed duration as the response variable. Our model included testing order as a covariate and individual identity as a random factor. In this analysis, we found that the behaviour did not vary among individuals (σ^2^ (SE) = 0.02 (0.20), χ^2^_0/1_=0.00, P=0.50). Furthermore, the behaviour exhibited no discernible dependence on partner identity (σ^2^ (SE)=0.00 (-), χ^2^_0/1_=0.00, P=0.50).
